# THE ROLE OF TREATMENT CHOICE IN CONFOUNDING ASSOCIATIONS BETWEEN HASHIMOTO AND ALZHEIMER’S DISEASE

**DOI:** 10.1093/geroni/igae098.2596

**Published:** 2024-12-31

**Authors:** Galina Gorbunova, Arseniy Yashkin, Stanislav Kolpakov Nikitin, Svetlana Ukraintseva, Anatoliy Yashin, Igor Akushevich

**Affiliations:** Duke University, Durham, North Carolina, United States; Duke University, Durham, North Carolina, United States; Duke University, Durham, North Carolina, United States; Duke University, Durham, North Carolina, United States; Duke University, Durham, North Carolina, United States; Duke University, Durham, North Carolina, United States

## Abstract

Alzheimer’s disease (AD) is a neurodegenerative disorder, most prevalent at the advanced ages. The increased risk of cognitive decline and the onset of AD and related dementia have recently been associated with hyperthyroidism while the results for hypothyroidism-AD association have been inconclusive. We hypothesized that hypothyroidism treatment modalities may cause variability in the available evidence of a hypothyroidism-AD association. We used Medicare administrative claims data from 2006-2022 to test this hypothesis by assessing how the association between Hashimoto’s disease, which is the most common form of hypothyroidism, and a clinical diagnosis of AD dementia changes as information about treatment modality is entered into the model. As was expected, non-treatment-enabled models were unable to find a statistically significant association between Hashimoto’s disease and AD. These findings were consistent across sex/gender and race/ethnicity-specific groups. The introduction of treatment modalities into the model, such as T-4 hormone replacement therapy, Liothyronine (and its generic equivalents), Levothyroxine (and its generic equivalents), surgery, and supplementary metformin use, modified the observed non-treatment-aware relationships. However, due to stratification, the prediction power of our treatment-aware models accounting for treatment modality was substantially weaker.

